# Interactions between the spike code and the epigenetic code during information processing in the brain

**DOI:** 10.3389/fnmol.2013.00017

**Published:** 2013-07-08

**Authors:** John Smythies, Lawrence Edelstein

**Affiliations:** ^1^Department of Psychology, Center for Brain and Cognition, University of California San DiegoLa Jolla, CA, USA; ^2^Medimark CorporationDel Mar, CA, USA

## Introduction

In a recent paper in this journal (Smythies and Edelstein, 2012) we described an epigenetic code involved in neurocomputation. This was based on the following facts. Clinical observations from a number of sources had shown that the modality of a sensory neuron (i.e., whether it is a visual or an auditory neuron) is determined by the origin of its afferent fibers. This can be changed if the afferent source is rerouted, and involves radical rebuilding of the functional neuroanatomy of the postsynaptic neuron. At that time the nature of the transsynaptic mechanism that effected this change was unknown. Our hypothesis suggested that the mechanism consists of the epigenetic material that contains the code (whose elements are protein transcription factors, DNA fragments and various types of RNA, including microRNAs) transported from the pre- to the post-synaptic neuron by exosomes. These exosomes enter the postsynaptic cell and some are transported to its nuclear zone (Waldenström et al., 2012). Neurons possess at least one other neurocomputational system—the familiar spike code mediated by electrochemical events. The spike code is fast and uses small molecules such as glutamate and acetylcholine as its effective agents. The epigenetic code is slower and uses protein synthesis as its effective agent. This rebuilds the hardware so as to process different inputs most effectively. The question then arises “How do these two codes interact?” This is one subject of this present paper. We also consider a second question “How do subsections of the epigenetic code interact with each other?” We end by considering the functional role of a part of the epigenetic code—DNA fragments carried by exosomes.

The first section relates what little is known of the interactions of microRNAs (major ingredients of the epigenetic code) with ion channels (major ingredients of the spike code) particularly in the nervous system.

## Section 1. actions of microRNAs upon ION channels

In recent experiments, microRNAs have been shown to modulate the subunit structure and the function of ion channels. In cardiac tissue microRNA expression regulates ion channel genes at the post-transcriptional level (Zhou et al., 2011). MicroRNAs induce alterations in expression and function of ion channels and transporters (Wang, 2010; Jiang et al., 2012). In cardiac tissue microRNA-301a modulates the potassium channel Kv4.2. (Panguluri et al., 2013). In the nervous system, a genome-wide association study of bipolar disorder Shih et al. (2012) found that many miRNA-targeted genes were functionally related to ion channels, collagen, and axonal growth and guidance that have been suggested to be associated with BPD previously. Some of these genes are linked in the literature to the regulation of miRNA machinery. The rhythmic expression of the microRNA gga-mir-26a regulates the protein expression and rhythmicity of the photoreceptor L-type voltage-gated calcium channel alpha1C subunit (L-VGCCalpha1C subunit) (Shi et al., 2009). Furthermore, a single microRNA (miR-103) simultaneously regulates the expression of the three subunits forming the L-type calcium channel (Cav1.2-LTC). MiR-103 knockdown in naive rats results in hypersensitivity to pain. This microRNA is down-regulated in neuropathic animals, whereas intrathecal injections of miR-103 relieve pain (Favereaux et al., 2011).

Thus the modulation by microRNAs of the structure and function of ion channels in the nervous system may provide a means for the interaction of the epigenetic code and the spike code. This is plausible since the spike code is heavily influenced by the characteristics of the ion channels that produce the potentials, in particular their precise relative timing (Zeng and Jung, 2004; Tiesinga et al., 2008; Cannon et al., 2010; Catterall et al., 2012), and would appear to add massively to the neurocomputational repertoire of the brain. Synaptic signals exquisitely modulate the production of microRNAs that in turn dynamically modulate their target mRNAs, so as to maintain a tight grip on the patterns of protein synthesis that is essential for learning and cognition. However, elucidation of the detailed mechanisms involved awaits further experiments.

## Section 2. interactions between DNA methylation and microRNAs

DNA methylation and microRNAs are key agents in the epigenetic code and interact at two levels. The level of DNA methylation controls the synthesis of microRNAs: and microRNAs modulate the activity of DNA methytransferase and DNA repair enzymes.

### MicroRNA—>DNA

– The proinflammatory cytokine IL-6 increases expression of DNA methyltransferase-1 (DNMT-1) and epigenetically regulates the expression of several genes, including those for the microRNAs (miRNAs) miR-148a, miR-152 that target DNMT-1 (Braconi et al., 2010).
– Down-regulated microRNA-152 induces aberrant DNA methylation in hepatitis B virus-related hepatocellular carcinoma cells by targeting DNA methyltransferase 1 (Huang et al., 2010).
– MicroRNA-152 mediates DNMT1-regulated DNA methylation in the estrogen receptor α gene (Wang et al., 2012).
– MicroRNA-450a targets DNA methyltransferase 3a in hepatocellular carcinoma (Weng et al., 2011).
– MicroRNA-152 targets DNA methyltransferase 1 in NiS-transformed cells via a feedback mechanism (Ji et al., 2013).
– MicroRNA-140 acts as a liver tumor suppressor by controlling NF-κB activity by directly targeting DNA methyltransferase 1 (Dnmt1) expression (Takata et al., 2013).
– During acute hyperglycemia miR-133a regulates DNA methylation by inhibiting Dnmt-1 (but not Dnmt-3a and -3b) methyltransferases in diabetic cardiomyocytes (Chavali et al., 2012).
– MicroRNA-342 inhibits colorectal cancer cell proliferation and invasion by directly targeting DNA methyltransferase 1 (Wang et al., 2012).
– MicroRNA-126 regulates DNA methylation in CD4+ T cells and contributes to systemic lupus erythematosus by targeting DNA methyltransferase 1 (Zhao et al., 2011).
– NA polymerase delta-interacting protein 38 is a target gene of microRNA-291a-5p (Lin and Lin, 2010).
– MicroRNA-143 targets DNA methyltransferases 3A in colorectal cancer (Ng et al., 2009).

### DNA —> miRNA

– MicroRNA-145 is regulated by DNA methylation and p53 gene mutation in prostate cancer (Suh et al., 2011).
– miR-152 is a tumor suppressor microRNA that is silenced by DNA hypermethylation in endometrial cancer (Tsuruta et al., 2011).
– A number of cancer-related genes are deregulated in bladder cancer at the levels of DNA methylation and mRNA expression, including genes HIC1, SLIT2, RASAL1, and KRT17 (Zhu et al., 2011).
– MicroRNA-219-2-3p Functions as a tumor suppressor in gastric cancer and is regulated by DNA methylation (Lei et al., 2013).
– MicroRNA-148a regulates runt-related transcription factor 3 gene expression via modulation of DNA methyltransferase 1 in gastric cancer (Zuo et al., 2013).
– MicroRNA miR-29c down-regulation leads to de-repression of its target DNA methyltransferase 3a resulting in ischemic brain damage (Pandi et al., 2013).
– MicroRNA-29b contributes to DNA hypomethylation of CD4+ T cells in systemic lupus erythematosus by indirectly targeting DNA methyltransferase 1 (Qin et al., 2012).
– Restoration of microRNA-214 expression reduces growth of myeloma cells through positive regulation of P53 and inhibition of DNA replication. (Misiewicz-Krzeminska et al., 2013).
– In a study of hepatocellular cancer, Shen et al. (2012) found multiple microRNAs that are downregulated by DNA methylation, which affects the host genes of miR-10a, miR-10b and miR-196b (HOXB4, HOXD4, and HOXA9, respectively).
– In a study of rheumatoid arthritis, de la Rica et al. (2013), using conjoint DNA methylation and miRNA screening, found sets of miRNAs that are controlled by DNA methylation, and genes (e.g., IL6R, CAPN8, and DPP4, as well as several HOX genes) that are regulated by DNA methylation and are targeted by miRNAs.
– In chronic lymphatic leukemia Baer et al. (2012), by integrating DNA methylation and miRNA promoter data, were able to identify 128 recurrent miRNA targets for aberrant promoter DNA methylation. DNA hypomethylation accounted for more than 60% of all aberrant promoter-associated DNA methylation in CLL, and promoter DNA hypomethylation was restricted to well-defined regions.
– Silencing of genes such as APC and CHFR, Sprouty 2, RASSF1A, GPR54, CDH1, and RSK4 by DNA hypermethylation, and regulation of gene expression by microRNAs may underlie the carcinogenic mechanisms of endometrial cancer (Banno et al., 2012).

### Miscellaneous

MicroRNAs also modulate the control of DNA repair and polymerase enzymes. For example:

– MicroRNA-182-5p targets a network of genes involved in DNA repair (Krishnan et al., 2013).
– DNA polymerase delta-interacting protein 38 is a target gene of microRNA-291a-5p (Lin and Lin, 2010)
– MicroRNA regulation of DNA repair gene expression occurs in hypoxic stress (Crosby et al., 2009).
– Germ cell apoptosis and death following exposure to xenoestrogens is orchestrated by miRNAs 29a, 29b, and 29c. This promotes increased protein levels of DNMT 1, 3a, and 3b concomitant with an increase in activity of the corresponding transcription level of their DNA target methylation genes L1td1-1 ORF1 and ORF2, Cdkn2a, and Gstp1 (Meunier et al., 2012).

Modulation of DNA methylation by microRNAs also occurs in embryogenesis e.g., during the transition from the morula to blastocyte (Lee et al., 2012).

## What is the function of extracellular and exosomal DNAs?

There have been several reports that exosomes contain fragments of DNA. In a comprehensive review Peters and Pretorius (2011) recognize that extracellular DNA exists inside exosomes and also as nucleoprotein complexes allowing horizontal gene transfer. Exosomes called prostasomes are secreted by the prostate and contain DNA fragments and the prostate specific membrane antigens (PSMA), CD38, and annexin A1 (Ronquist et al., 2012). In a study of cardiomyocytes, Waldenström et al. (2012), detected 343 different chromosomal DNA sequences in their microvesicles/exosomes. They observed microvesicle/exosomal DNA transfer into target fibroblasts, where exosomes stained for DNA were seen in the fibroblast cytosol and even in the nuclei. Astrocytes and glioblastomas release exosomes that contain mitochondrial DNA (Guescini et al., 2010). The most significant report comes from Cai et al. (2013) who detected at least 16,434 genomic (gDNA) fragments from extracellular vesicles (exosomes) obtained from human plasma. Immunofluorescence showed that these exosomes could be transferred into cells and localize to and inside the nuclear membrane. Here further tests showed that these fragments were composed of double stranded DNA (6–17 kb i.e., 6000–17,000 base pairs), did not derive from apoptosis, and were physiologically fully active and able to generate functional mRNAs.

The question is what could be the function of these large fragments of DNA transmitted from one cell to another? It is possible that the answer may lie in the field of transposons or ‘jumping genes’. *Mariner* transposons (transposable elements Class II) are lengths of DNA that migrate between individuals even of different species (Robertson et al., 1999). The carrier may by DNA viruses. Retrotransposons (transposable elements class I) are segments of chromosomal DNA that are transcribed into segments of RNA. These migrate to a different part of the genome, and there are converted by a reverse transcriptase back into DNA and reinserted into the gene. The function of both classes is at present obscure. Kim et al. (2012) say no more than that they may cause “genomic and genetic variations”. We suggest that some of the RNA exported by exosomes may be part of the retrotransposon system, and the DNA transported by exosomes may represent part of the *mariner* transposon system.

In a recent review Noble (2013) comments on retrotransposons and transposons as follows.

“Retrotransposons are DNA sequences that are first copied as RNA sequences, which are then inserted back into a different part of the genome using reverse transcriptase. DNA transposons may use a cut and paste mechanism that does not require a RNA intermediate …. DNA transposons insert into the genome in a functionally significant way …. We now know that animal genomes are full of transposons …. An important point to note is the functionally significant way in which this communication can occur. In bacteria, starvation can increase the targeted transposon-mediated reorganizations by five orders of magnitude, i.e. by a factor of over 100,000.”

So far these systems have been considered only as functioning between organisms (transposons) and within single cells (retrotransposons). We suggest that the exosome system may provide a route for this type of intercellular genetic communication.
